# Labdanum Resin from *Cistus ladanifer* L. as a Source of Compounds with Anti-Diabetic, Neuroprotective and Anti-Proliferative Activity

**DOI:** 10.3390/molecules29102222

**Published:** 2024-05-09

**Authors:** David F. Frazão, Carlos Martins-Gomes, Teresa Sosa Díaz, Fernanda Delgado, José C. Gonçalves, Amélia M. Silva

**Affiliations:** 1 Center for Research and Technology of Agro-Environmental and Biological Sciences (CITAB-UTAD), University of Trás-os-Montes e Alto Douro (UTAD), Quinta de Prados, 5001-801 Vila Real, Portugal; davidmfrazao@gmail.com (D.F.F.); camgomes@utad.pt (C.M.-G.); 2 Plant Biotechnology Center of Beira Interior (CBPBI), Quinta da Senhora de Mércules, Apartado 119, 6001-909 Castelo Branco, Portugal; fdelgado@ipcb.pt (F.D.); jcgoncalves@ipcb.pt (J.C.G.); 3 Mediterranean Institute for Agriculture, Environment and Development (MED), Centre of Agronomic and Agro-Industrial Biotechnology of Alentejo (CEBAL), 7801-908 Beja, Portugal; 4 Department of Plant Biology, Ecology and Earth Sciences, Faculty of Science, University of Extremadura, 06006 Badajoz, Spain; tesosa@unex.es; 5 Research Centre for Natural Resources, Environment and Society (CERNAS), Polytechnic Institute of Castelo Branco (IPCB), Quinta da Senhora de Mércules, Apartado 119, 6001-909 Castelo Branco, Portugal; 6 Department of Biology and Environment, School of Life Sciences and Environment, University of Trás-os-Montes and Alto Douro (UTAD), Quinta de Prados, 5001-801 Vila Real, Portugal

**Keywords:** α-amylase, α-glucosidase, acetylcholinesterase, cytotoxic activity, labdanum resin, labdane-type diterpenoids, methylated flavonoids

## Abstract

Labdanum resin or “gum” can be obtained from *Cistus ladanifer* L. by two different extraction methods: the Zamorean and the Andalusian processes. Although its main use is in the fragrance and perfumery sectors, ethnobotanical reports describe its use for medicinal purposes in managing hyperglycemia and mental illnesses. However, data concerning the bioactivities and pharmacological applications are scarce. In this work, it was found that the yield of labdanum resin extracted by the Andalusian process was 25-fold higher than the Zamorean one. Both resins were purified as absolutes, and the Andalusian absolute was purified into diterpenoid and flavonoid fractions. GC-EI-MS analysis confirmed the presence of phenylpropanoids, labdane-type diterpenoids, and methylated flavonoids, which are already described in the literature, but revealed other compounds, and showed that the different extracts presented distinct chemical profile. The potential antidiabetic activity, by inhibition of α-amylase and α-glucosidase, and the potential neuroprotective activity, by inhibition of acetylcholinesterase, were investigated. Diterpenoid fraction produced the higher α-amylase inhibitory effect (~30% and ~40% at 0.5 and 1 mg/mL, respectively). Zamorean absolute showed the highest α-glucosidase inhibitory effect (~14% and ~24%, at 0.5 and 1 mg/mL, respectively). Andalusian absolute showed the highest acetylcholinesterase inhibitory effect (~70% and ~75%, at 0.5 and 1 mg/mL, respectively). Using Caco-2 and HepG2 cell lines, Andalusian absolute and its purified fractions showed moderate cytotoxic/anti-proliferative activity at 24 h exposure (IC_50_ = 45–70 µg/mL, for Caco-2; IC_50_ = 60–80 µg/mL, for HepG2), whereas Zamorean absolute did not produce cytotoxicity (IC_50_ ≥ 200.00 µg/mL). Here we show, for the first time, that labdanum resin obtained by the Andalusian process, and its fractions, are composed of phytochemicals with anti-diabetic, neuroprotective and anti-proliferative potential, which are worth investigating for the pharmaceutical industry. However, toxic side-effects must also be addressed when using these products by ingestion, as done traditionally.

## 1. Introduction

The rockrose *Cistus ladanifer* L. (Cistacea family, subgenus *Leucocistus*, Ladanium section), namely the subspecies *ladanifer*, is an abundant and widespread plant resource in the Iberian Peninsula, including Portugal and Spain [1,2,3]. A shrubland dominated by *C. ladanifer* tends to persist rather than to develop into mature vegetation stage and, in addition, presents low abundance of other plant species [4,5], registering their occupation mostly on abandoned and degraded areas. It is, therefore, a not-endangered plant resource worth being exploited in a sustainable manner.

Currently, the most significant valorization of such resource is the extraction of its exuded resin (which has the commercial name of labdanum “gum”) for the fragrance/perfumery industry, because of its aromatic and fixative properties, replacing ambergris used to be obtained from the protected sperm whale *Physeter catodon* [6]. In fact, according to *Biolandes* SAS (Le Sen, France), one of the most important producers of *C. ladanifer* extracts, around 300–350 tons of raw labdanum resin are extracted each year from 6000–7000 tons of manually harvested plants [7]. To diversify and increase the economy around this plant, in addition to perfumery, it is necessary to find other applications for labdanum resin. This diversification is extremely relevant to increasing management of these agro-forestry systems.

Traditionally, labdanum resin is extracted, using water, by the Zamorean process or the Andalusian process [8]. The Zamorean process is a physical extraction where labdanum resin is removed from the surface of *C. ladanifer* plant material using boiling water, and then the resin at the surface of the water is continuously separated using a skimmer [8]. The Andalusian process is a chemical extraction where the labdanum resin is extracted from the surface of the plant material with warm alkaline water and, afterwards, precipitated by lowering the pH with acid and, finally, separated with a skimmer or by decantation [8]. Labdanum resin is mostly composed by labdane-type diterpenes and methylated flavonoid aglycones [9,10]. To separate epicuticular lipids, mostly waxes, from the labdanum, resin and to obtain purified diterpenoid and flavonoid fractions, a cold methanol precipitation followed by molecular sizing column chromatography has been used [9,10,11,12,13,14,15].

Concerning medicinal applications, ethnobotanical studies report that tisanes or infusions of labdanum or *C. ladanifer* shoots and leaves were traditionally ingested by humans as medicine to heal disorders of the gastro-intestinal system (e.g., toothaches, stomach ache, peptic ulcers, colon pain and liver disorders), respiratory system (e.g., colds and whooping cough), nervous system and mental illness (e.g., sedative for anxiety, insomnia, and digestive spasms, and neuralgia), and to control blood sugar spikes and rheumatism, besides the extensive use to heal skin disorders [6,16,17,18,19,20]. The traditional use of *C. ladanifer* shoot and leaf “aqueous extracts” is much more reported than the use of labdanum.

Recently, labdanum resin was revealed to have high anti-inflammatory activity, mainly due to the content in flavonoid aglycones, a property assessed by the inhibition of nitric oxide release by LPS-stimulated RAW 264.7 cells (a macrophage cell line), and also revealed antioxidant properties [21]. This activity may explain several traditional medicinal applications enumerated above.

Given the traditional use of *Cistus* spp. to control hyperglycaemia, aqueous and hydromethanolic extracts from the Marroccan *Cistus salviifolius* L. and *Cistus monspeliensis* L., were reported to moderately inhibit α-amylase and strongly inhibit α-glucosidase activity [22], these enzymes are relevant in the management of diabetes mellitus type 2 by reducing carbohydrate breakdown and subsequent absorption [20]. However, there is no information regarding the antidiabetic effect *C. ladanifer* labdanum and, thus, it would be worth studying *C. ladanifer* labdanum’s inhibitory activity on these enzymes to evaluate its anti-diabetic potential. Likewise, given the documented traditional use of *Cistus* spp. to control nervous system and mental disorders [23,24], studying the inhibitory action of *C. ladanifer* labdanum on acetylcholinesterase (AChE) activity is relevant. In fact, cholinesterases’ inhibition, AChE and, to some extent butyrilcholinesterase (BChE), are the current therapeutic strategies to manage neurodegenerative disorders such as Alzheimer’s disease by directly improving cholinergic tone (increasing acetylcholine neurotransmitter) and by the less understood long-term anti-neurodegenerative activity [25]. 

However, given that labdanum resin, or preparations from it, are in some medicinal applications intended to be ingested, there is a need to assess their toxicity, or safety profile, at gastrointestinal and hepatic level. Thus, as a preliminary approach, the assessment of in vitro cytotoxic activity using relevant cell models, such as human colorectal adenocarcinoma (Caco-2) and human hepatocyte carcinoma (HepG2) cell lines was used to screen potential toxicity of the resin, or of its extracts, to the gastrointestinal epithelial barrier and to the hepatocyte cells, respectively.

Therefore, the main aims of this study were to assess the anti-diabetic and neuroprotective effect of the labdanum resin, and of its extracts, by evaluating the inhibition of relevant enzymes, and to evaluate the cytotoxic/safety profile of these extracts using relevant cell lines. Thus, we show for the first time that labdanum resin has antidiabetic and neuroprotective properties. And, we identified a range of safe concentrations that can be considered when using it orally for medicinal purposes. This study establishes a starting point for future studies using more complex in vitro and in vivo models, aiming to develop new pharmaceutical formulations based on this natural resource.

## 2. Results and Discussion

### 2.1. Extraction Yields and Chemical Characterization of Labdanum and Fraction Extracts

In this study, labdanum resin, extracted according to the Andalusian process (Adl), yielded 5.79 ± 0.52% (dw/fw) (Table 1). The Zamorean process (Zam) was performed as a control to evaluate the importance of alkaline extraction of labdanum resin. In fact, the Zam extraction yielded only 0.23 ± 0.07%, which is approximately 25 times lower than the yield of Adl resin. In this study, Zam resin was successfully isolated, however, using acidification to precipitate it, as was done for the Adl resin. Despite the difference of extraction yields, both resins rendered similar absolute yields of around 70% (dw/dw). Diterpenoid and flavonoid fractions were obtained by column chromatography from the Adl labdanum absolute, rendering 79.86 ± 0.76% and 10.64 ± 2.17% (dw/dw) yields, respectively. A remainder fraction, lost during the purification process, represented 9.48 ± 1.87% (dw/dw) of the absolute. As the yield of Zam labdanum resin was too low, it was not fractionated.

The chemical profile of the labdanum resins and purified fractions was assessed by GC-EI-MS with a prior TMS derivatization reaction of the mixtures. Fragment ion 73 *m*/*z* was used to confirm TMS derivatives [26]. Overall, chromatograms of extracts revealed 37 peaks (Figures S1–S4) with a peak area above 2% in relation to the major peak, which depended on the extract (indicated in Table 2). Using standards, eight peaks could be identified as typical labdane-type diterpenoids or flavonoid aglycones. Using the NIST library, only 3-phenylpropanoid acid compound could be identified as a TMS derivative, with an excellent fragmentation pattern match of 921 (peak 2, 9.64 min). Other compounds were identified using NIST library with a good match (800–900); however, most were methyl ester derivatives. Those compounds, identified as methyl ester derivatives, presented a significant amount of ion (73 *m*/*z*), which means that they are most likely TMS derivatives. Interestingly, carboxylic acids (phenylpropanoids, fatty acids and diterpenoid acids) presented ion at 73 *m*/*z,* but alcohols (rhododendrol and flavonoids) did not, which is inconsistent with what is described by Harvey and Vouros [26].

As observed in Table 2, the diterpenoid fraction (Figure S2) was shown to be mostly composed by labdane-type diterpenoid acids, the flavonoid fractions (Figure S3) were shown to be mostly composed by methylated flavonoid aglycones with apigenin and kaempferol skeleton, and the Adl labdanum absolute (Figure S1) was shown to be mostly composed by labdane-type diterpenoid acids, fatty acids, and jaranol. Zam labdanum absolute (Figure S4) was shown to be mostly composed of phenylpropanoids, fatty acids, and several non-identified compounds, and to have a different chemical profile to the Adl labdanum absolute.

### 2.2. Evaluation of Anti-Diabetic and Neuroprotective Potential of Labdanum and Fraction Extracts

Concerning the anti-diabetic activity, inhibition of the enzymes involved in the starch breakdown, which reduces glucose availability to intestinal absorption, was assessed. Inhibition of α-amylase and α-glucosidase activity by labdanum absolutes and purified fractions is presented in Table 3 and Table 4, respectively. Diterpenoid fraction was the extract with the highest α-amylase inhibitory activity (around 40 and 30% inhibition at 1 and 0.5 mg/mL, respectively) followed by Adl labdanum absolute. Flavonoid fraction presented low α-amylase inhibitory capacity (~4% at 1 mg/mL), and Zam labdanum absolute did not inhibited the enzyme (Table 3). In contrast (Table 4), Zam labdanum absolute produced the highest inhibitory effect on α-glucosidase activity (around 25% at 1 mg/mL), followed by Adl labdanum absolute (~14% inhibition at 1 mg/mL) and flavonoid fraction, which showed similar inhibitory effect. Diterpenoid fraction showed the lowest capacity to inhibit α-glucosidase activity (~6.5% inhibition, at 1 mg/mL).

Comparing Dit and Flv fractions, while the Dit fraction presented higher potential in inhibiting α-amylase, the Flv fraction presented higher potential in inhibiting α-glucosidase. Of the compounds identified in Table 2, apigenin, genkwanin and acacetin have been tested for their anti-α-amylase and anti-α-glucosidase activities [27]. In fact, apigenin and genkwanin produced higher α-glucosidase inhibition when compared to α-amylase, supporting the results presented here (Table 3 and Table 4). Acacetin showed similar inhibitory activity for both enzymes [27].

A variety of fungi- and plant-based metabolites belonging to several classes of compounds, including terpenoids and flavonoids, have shown α-amylase and α-glucosidase inhibitory activity [20,28,29]. Acarbose is currently one of the drugs used to control hyperglycemia in individuals with type-2 diabetes by competitively and reversibly inhibiting intestinal α-glucosidases, a family of enzymes that hydrolyzes oligosaccharides into glucose and other monosaccharides, and non-reversibly inhibiting α-amylase, secreted by salivary glands and exocrine pancreas, that cleave complex polysaccharides (e.g., starch) into oligosaccharides [20,30]. We observed that the acarbose inhibitory effect was higher against α-amylase (~75% and ~87% inhibition at 0.5 mg/mL and 1 mg/mL, respectively) than against α-glucosidase (~70% and ~80% inhibition at 0.5 mg/mL and 1 mg/mL, respectively); this inhibitory trend is reported by other authors (e.g., [31]).

Labdanum absolutes and fractions are not so effective in inhibiting α-amylase (Table 3) or α-glucosidase (Table 4) as acarbose; however, this extract and fractions have high potential as anti-diabetic agents, since the inhibitory effect against α-amylase and α-glucosidase is much higher than that of medicinal and aromatic plants commonly reported to have antidiabetic activities. These extracts (Table 3 and Table 4) show higher or similar antidiabetic activity than *Thymus pulegioides* L. aqueous or hydroethanolic extracts (10% inhibition of α-glucosidase and none effect at α-amylase, at 0.5 mg/mL extracts) [28], or orange thyme extracts that at 0.5 mg/mL inhibited 10% of α-amylase and 12% of α-glucosidase activity [32].

Concerning *Cistus* spp., hydromethanolic extracts obtained from *Cistus salviifolius* L. and *Cistus monspeliensis* L. aerial parts showed very high α-glucosidase (IC_50_ ~0.01 mg/mL, for both plants) activity and high α-amylase (IC_50_ ~0.6 and 0.7 mg/mL, for *C. salviifolius* and *C. monspeliensis*, respectively) activity [22]. Aqueous infusion extracts of *C. ladanifer* were reported to inhibit α-amylase (IC_50_ ~1.3 mg/mL); however, these inhibitory assays [33] were performed at different temperature conditions from the ones reported here. These results indicate that labdanum resin, in particular the diterpenoid fraction, has compounds with α-amylase activity (Table 3), while the flavonoid fraction has compounds with higher activity against α-glucosidase (Table 4).

AChE inhibition activity of labdanum absolutes and purified fractions is shown in Table 5. Adl labdanum absolute showed the highest inhibition effect (~70% and 75% inhibitory activity, at 0.5 and 1 mg/mL, respectively), but Zam labdanum absolute only produced ~22% and 7% AChE inhibitory effect at 1 and 0.5 mg/mL, respectively. Adl labdanum fractions showed moderate AChE inhibitory activities, indicating that compounds present in both fractions have AChE target ability. Around 10% (dw/dw) of the Adl labdanum absolute was lost during the purification of diterpenoid and flavonoid fractions. GC-EI-MS analysis revealed that there are compounds present in significant amounts in Adl labdanum absolute, which are not present in both purified fractions (between peak 13, at 26.48 min, and peak 31, at 39.99 min, Table 2); this may explain the higher activity of the absolute comparing to the sum of fractions activity. Several plant extracts and plant-derived compounds have been shown to possess AChE inhibition activity, mostly alkaloid compounds [34], and phenolic rich extracts, such as the aqueous and hydroetanolic extracts of *T. pulegioides* that, at 0.5 mg/mL, inhibited ~82% and 89% AChE, respectively [28]. Concerning Cistus spp., essential oils of five species (*Cistus creticus*, *Cistus salviifolius*, *Cistus libanotis*, *Cistus monspeliensis* and *Cistus villosus*) were reported to have AChE inhibitory activity with wide range of activity (at 0.5 mg/mL ~12% and ~90% inhibition for *C. creticus* and *C. libanotis*, respectively) [35]. In this work, we report, for the first time, the inhibition of AChE by a *C. ladanifer* extract, the labdanum resin (absolute and fractions) and, thus, its neuroprotective potential.

Furthermore, since alkaloids are not reported for this species, this activity may be associated with compounds belonging to the flavonoid or terpenoid classes. In fact, compounds belonging to these classes of phytochemicals have shown promising potential as AChE inhibitors [36,37]. Research on the inhibitors of AChE is important for the development of drugs with neuroprotective activity, since decreased (or altered) cholinergic function is observed in many neurodegenerative disorders, such as Alzheimer’s disease, senile dementia, Parkinson’s disease, and others [25]. Rivastigmine (based on physostigmine chemical structure, from *Physostigma venenosum*) and galanthamine (an alkaloid from *Galanthus woronowii*) are among the most prescribed cholinergic enhancers, acting as AChE inhibitors, and are derived from natural sources [38]. Finding new AChE inhibitors is, thus, essential for the development of new pharmaceutical drugs. It is also relevant, but to a lesser extent, for the development of useful toxins for agriculture, principally those selectively toxic for arthropods and not to vertebrates [25,39].

The anti-AChE activity has also been previously described for some of the compounds identified in Table 2, or for similar compounds. Octadecanoic acid, whose methyl ester and hydropropyl ester derivatives were identified in Adl and/or Zam extracts, was shown to inhibit AChE activity [40]. Regarding flavonoid derivatives, Jurčević Šangut et al. [27] reported the anti-AChE activity of apigenin, acacetin and genkwanin, all identified in Flv fraction (Table 2). In addition, hydroxycinnamic acids were also shown to be potential neuroprotective agents through the inhibition of AChE activity.

### 2.3. Anti-Proliferative/Cytotoxic Activity of Labdanum Absolutes and Fractions

**Figure 1:**
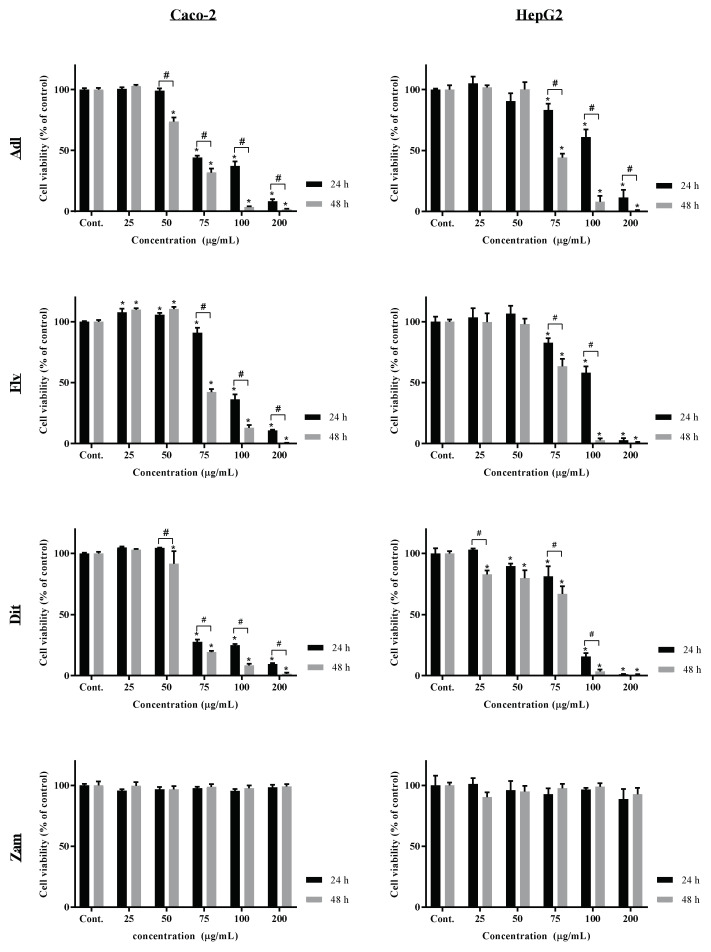
The effect of labdanum resin extracted by the Andalusian process (Adl), its purified flavonoid (Flv) and diterpenoid extracts (Dit), and labdanum resin extracted by the Zamorean process (Zam) on Human epithelial colorectal adenocarcinoma (Caco-2) and Human hepatocellular carcinoma (HepG2) cells viability (% of control) after 24 and 48 h incubation (3 independent experiments of *n* = 4 each). * means statistical differences in relation to the control by the post hoc Tukey’s test (α = 0.05). # means statistical differences between incubation times by the post hoc Tukey’s test (α = 0.05).

Anti-proliferative or cytotoxic activity of labdanum absolutes and fractions was performed against two relevant cell lines, HepG2 (human hepatocellular carcinoma cell line) and Caco-2 (human colorectal adenocarcinoma cell line), namely if medicinal preparations are aimed for ingestion purposes. Caco-2 cells represent the absorptive enterocytes, and HepG2 cells the first pass hepatocytes. Caco-2 and HepG2 cells were exposed to different concentrations of both labdanum absolutes (Adl and Zam absolutes) and of Adl fractions (diterpenoid and flavonoid fractions), for 24 h and 48 h; the results are shown in Figure 1, and the calculated IC_50_ values (i.e., the concentration that inhibits 50% of cell viability/proliferation) are shown in Table 6. Sigmoid curves of HepG2 and Caco-2 cells viability data versus extracts concentration are presented in Figures S5 and S6, respectively. The Hill slope parameter and the R^2^ of the sigmoid curves are presented in Table S1. As shown in Figure 1, Zam absolute induced no cytotoxicity at the concentrations tested (25–200 µg/mL), so the sigmoid regressions could not be projected. Regarding the other extracts, sigmoid regressions presented a R^2^ higher than 0.9 at all conditions, considering them a good fit to extract the IC_50_ parameter.

As observed in Figure 1, Zam labdanum absolute did not show cytotoxic activity against both cell lines at the maximum tested concentration (200 µg/mL). On the other hand, Adl labdanum resin and its purified diterpenoid and flavonoid fractions presented a dose-dependent cytotoxicity against both cell lines. At 100 µg/mL, Adl labdanum resin and fractions reduced cell viability to values below 30% of control, independently of the cell line, incubation time, and extract.

Regarding the IC_50_ parameter, it is evident that Caco-2 cells are more susceptible than HepG2 cells to all extracts. In general, at 24 h exposure, the diterpenoid purified fraction presented on average higher cytotoxicity against Caco-2 and HepG2 cells, as indicated by the lower IC_50_ values (Table 6). Although at 48 h incubation, the cytotoxic activity of Adl labdanum resin was on average higher (lower IC_50_), against both cell lines, the cytotoxic activity was not statistically different (α > 0.05) from the flavonoid and diterpenoid for Caco-2 cells, or from the diterpenoid for HepG2 cells and fractions. The Caco-2 cell line used is commonly used as a model of the intestinal epithelial barrier, because it retains the typical properties of absorptive enterocytes [41], and the HepG2 cell line is used as a model to predict hepatotoxic potential of substances because hepatocyte features and properties are maintained [42]. Given the results observed in this study, labdanum resin is potentially toxic to the gastrointestinal tract if ingested and to the liver if absorbed. However, having in account that these are cancer cell lines, this cytotoxic/anti-proliferative activity could be regarded as a cytostatic action and, thus, these extracts and their individual compounds (e.g., methylated flavonoid aglycones and labdane-type diterpenoids) are worth being studied in the future as anti-cancer agents.

To the best of our knowledge, this is the first work reporting the anti-proliferative activity of *Cistus ladanifer* labdanum resin absolute and of its purified fractions. However, Barrajón-Catalán et al. [43] reported the cytotoxicity effect of a hot-water extract from the milled shoots of *C. ladanifer* against several human cancer cell lines, showing IC_50_ values between 0.49 and 16.10 mg/mL, much higher than those obtained in this study (which were lower than 0.1 mg/mL). These differences might be related to the extract composition, as hot-water extracts are rich in polyphenols, flavonoids and tannins [43], and labdanum (Table 2) is also rich in diterpenoids, which enhance cytotoxicity [44]. Gaweł-Bęben et al. [45] reported that *C. ladanifer* aerial parts extracted with methanol (at different proportions with water) rendered extracts with cytotoxic activity against A375 (human malignant melanoma), with IC_50_ values between 100 and 200 µg/mL, depending on the extract, but were ineffective against human squamous cell carcinoma (ACC-15) cells; IC_50_ > 500 µg/mL, higher values than the results shown in this study. *Cistus ladanifer* extracts (acetone/water and ethanol) only slightly affected skin fibroblast viability [46]. Extracts from other *Cistus* spp. were reported to have anti-proliferative activity, as is the case of the ethanol extract of *Cistus creticus* subsp. *creticus* L. shoots that was cytotoxic on cervix carcinoma (HeLa), breast apocrine carcinoma (MDA-MB-453) and melanoma (FemX) cancer cells, with IC_50_ reaching 80.83 μg/mL, 76.18 μg/mL, and 87.52 μg/mL, respectively [47], values that are identical to those obtained in HepG2 cells at 24 h exposure (Table 6). *Cistus incanus* L. extracts, obtained with different organic solvents, rich in polyphenols were tested against human colon adenocarcinoma (HCT116) cells, showing IC_50_ values between ~140 μg/mL and 400 μg/mL, depending on the extract [48] that, although not so effective as the extracts obtained here (Table 6), corroborates the anti-proliferative activity against colon cancer. This comparison with the literature suggests that Adl labdanum absolute, but not Zam labdanum absolute, has higher cytotoxic activity than *C. ladanifer* whole plant extracts (water or organic solvent extracts). It is worth emphasizing that, considering the IC_50_ values obtained here, and according to NCI guidelines, Adl extracts and fractions are considered moderately active (IC_50_ 0.02–0.20 mg/mL) as anti-cancer agents.

Therefore, in this study, we provide a first assessment of labdanum resin bioactivities using in vitro enzymatic and cell-based assays. While the resin and its fraction present anti-diabetic, neuroprotective and anti-proliferative potential, these results should be confirmed in a broader set of cell models, as well as in more complex experimental models, such as 3D cultures and in vivo models. These would allow us to confirm both their pharmacological potential and their safety profile.

## 3. Materials and Methods

### 3.1. Labdanum Resin Extraction and Purification

*Cistus ladanifer* subsp. *ladanifer* shoots were collected from a shrubland in Penha Garcia, Portugal (GPS: N 40°1′43.4″ W 6°59′34.8″), in August of 2018. Plant material was stored fresh in the freezer (−20 °C) until use.

To obtain labdanum resin, two different methods were used: the Andalusian process (Adl) and the Zamorean process (Zam). In Adl process, 25 g of plant material were extracted using 200 mL of Na_2_CO_3_ aqueous solution (25 g/L). The extraction was carried out in a flask, at atmospheric pressure, for 1 h at 60 °C (water bath). The solution was then filtered through a stainless-steel sieve (mesh n° 140, 106 µm aperture). After cooling, a H_2_SO_4_ solution (5 M) was added dropwise to the extract, under agitation, until reaching pH 2. The precipitated resin was separated from the supernatant by centrifugation, at 3030× *g* for 15 min (Mega Star 600R, VWR; Alfragide, Portugal), and finally freeze-dried [21].

To simulate the Zamorean process (Zam) [8], labdanum resin was extracted by using the same procedure described above, but using distilled water instead of a Na_2_CO_3_ aqueous solution. All extractions were done in triplicate, resulting on three similar resins (*n* = 3). The resin yield of the extractions was calculated by the ratio between the total dry weight (dw) of the resin and the fresh weight (fw) of the plant material (presented in %).

Labdanum absolute was obtained by removing “waxes” from the resins by dissolving 1 g of the resins in 20 mL of methanol under an ultrasonic bath. Then, the solution was left at −20 °C overnight, and afterwards centrifuged at 3030× *g* at −5 °C for 15 min. The procedure was repeated three times, and supernatants were joined as the absolute. This method was first described by Vogt, Proksch and Gülz [14], and used in recent works [9,10,15].

Diterpenoid (Dit) and Flavonoid (Flv) purified fractions were obtained by eluting the Adl labdanum resin on Sephadex^TM^ LH-20 (GE Healthcare Biosciences AB; Uppsala, Sweden) compacted in a 25 cm long and 1.5 cm wide column, using methanol as eluent (the Zam labdanum resin was not fractionated due to low yield). Sub-fractions of 2–3 mL were continuously collected and analyzed by thin layer chromatography using silica gel TLC plates (F254s 0.2 mm 10 × 20 cm) (Merck KGaA, Darmstadt, Germany). Sub-fractions were eluted with toluene: ethyl acetate (9:1). Flavonoid bands were visualized at 312 nm using a trans-illuminator. After being sprayed with 10 M H_2_SO_4_ solution and heated at 100 °C for 10 min in a ventilated chamber, diterpenoid bands were visualized as purple/red bands. Sub-fractions were joined to make the purified fractions.

### 3.2. Gas Chromatography Coupled to an Electron Ionization Mass Spectrometer (GC-EI-MS)

Labdanum absolutes and fractions were prepared at 10 mg/mL. A volume of 20 µL of BSTFA (*N*,*O*-bis(trimethylsilyl)trifluoroacetamide) + 10% TMCS (trimethylsilyl chloride) was added to 100 µL of sample for derivatization, at room temperature, during 30 min, according to Azemard et al. [49]. A volume of 1 µL of the reaction mixture was injected at a split ratio of 1:50 in the gas chromatography system equipped with a HP5-MS column (30 m × 0.25 mm × 0.25 µm, J&W GC columns; Agilent, Santa Clara, CA, USA). Helium was used as carrier gas at 1 mL/min flow rate. Temperature gradient was set as follows: 50–175 °C (10 °C/min), 175–300 °C (3 °C/min). Data was acquired through electron impact ionization mass spectrometry (EI-MS, scan mode of positive *m*/*z* ions 50–650, 70 eV ionization potential). Injector and detector port temperatures were set at 250 °C. The major peaks of the sample chromatogram were tentatively identified using the NIST library (2017) mass spectra and Kovats retention index database, and by matching their retention times and mass spectra to standards: Labdanolic acid and 6-oxocativic acids were acquired from Merck (Darmstadt, Germany); apigenin, kaempferol, kaempferol-3-methyl ester, kaempferol-3,4′-dimethyl ester, apigenin-4′-methyl ester, apigenin-7-methyl ester, apigenin-7,4′-dimethyl ester, kaempferol-3,7-dimethyl ester, and kaempferol-3,7,4-trimethyl ester were acquired from Carbosynth Lda, (Berkshire, UK).

### 3.3. α-Amylase, α-Glucosidase, and Acetylcholinesterase (AChE) Inhibition

All enzymatic inhibition assays were performed in 96-well microplates and absorbances were read using a microplate spectrophotometer (Multiskan EX microplate reader (MTX Labsystems; Bradenton, FL, USA)). Methods were performed as described in Martins-Gomes et al. [50]. Labdanum absolutes and fractions (further denominated as samples) were diluted in PBS (at 0.5 and 1 mg/mL) from stock solutions prepared in DMSO. The final concentration of DMSO was always less than 5%, and a control with 5% DMSO was used in each assay to exclude solvent-induced inhibition. All the assays were done in triplicate (*n* = 3).

α-Amylase inhibition assay: 35 µL of PBS and 35 µL of starch solution (0.05% *m*/*v*, pH 7) were added to 50 µL of the samples. The mixtures were incubated for 2 min at 37 °C, and 20 µL of α-amylase (50 µg/mL in 10 mM phosphate buffer, pH 6.9) was added. After incubation for 10 min at 37 °C, 50 µL of HCl (0.1 M) was added to stop the reaction. Afterwards, 150 µL of Lugol solution (0.5 mM I_2_, 0.5 mM KI, in water) was added and, after a 15 min incubation, absorbance was read at 580 nm.

α-Glucosidase inhibition was assessed by first obtaining a crude enzyme extract by diluting 250 mg of rat’s intestinal acetone powder in 10 mL of phosphate buffer (0.1 M, pH 7), centrifuging for 20 min at 4 °C (5000× *g*), and recovering the supernatant. Then, 100 µL of the supernatant was joined to 50 µL of the samples and left to incubate for 10 min at room temperature. Afterwards, 50 µL of *p*-nitrophenyl-α-D-glucopyranoside (5 mM in phosphate buffer 0.1 M, pH 7) was added and, after a 30 min incubation at 37 °C, absorbance was read at 405 nm.

Acetylcholinesterase (AChE) inhibition was assessed by first adding 125 µL of Ellman’s reagent (0.3 mM DTNB, prepared in 50 mM Tris-HCl, pH 8.0), and 25 µL of acetylthiocholine iodide (1.5 mM, in 20 mM Tris-HCl, pH 7.5) to 50 µL of the samples. The mixtures were incubated for 2 min, and then 25 µL of AChE (0.026 U/mL in 20 mM Tris-HCL, pH 7.5) was added. After incubation for 10 min at 25 °C, absorbance was read at 405 nm.

Blanks including samples without the enzymes, controls including the enzymes without any sample, and positive control including acarbose (α-glucosidase and α-amylase; 1 mg/mL acarbose inhibited ~87% of α-amylase and ~80% α-glucosidase activity, at indicated experimental conditions) were performed. The blanks’ absorbance values were subtracted from the sample value. The percentage of inhibitions were calculated according to Equation (1), except for α-amylase which was calculated according to Torres-Naranjo et al. [51].

(1) $$Inhibition\;\left(\%\right)=\frac{\left({Abs}_{control}-{Abs}_{sample}\right)}{{Abs}_{control}}\times 100$$

### 3.4. Caco-2 and HepG2 Cell Cytotoxicity Assay

The cytotoxicity/anti-proliferative assay was performed in Human epithelial colorectal adenocarcinoma (Caco-2; Cell Lines Service (CLS), Eppelheim, Germany) and in Human hepatocellular carcinoma (HepG2; ATCC^®^ N.: HB-8065TM, ATCC, Rockville, DC, USA). Cell maintenance and handling were performed as described by Silva et al. [52]. Briefly, cell cultures were maintained in Dulbecco’s Modified Eagle Medium (DMEM) supplemented with 10% (*v*/*v*) fetal bovine serum (FBS), antibiotics (100 U/mL of penicillin and 100 μg/mL of streptomycin) and 1 mM L-glutamine in an atmosphere of 5% (*v*/*v*) CO_2_/95% (*v*/*v*) air, at 37 °C with controlled humidity.

Cells were detached from the culture flasks with trypsin-EDTA and, after counting (TC10™ Automatic counter, BIORAD; Amadora, Portugal), were diluted to a density of 5 × 10^4^ cells/mL (in complete culture medium), and then seeded in 96-well microplates (100 µL/well; 5 × 10^4^ cells/mL). Cells were allowed to adhere and stabilize for 48 h, after which the culture media was removed and replaced with 100 µL of sample solutions diluted in FBS-free culture media (range 6.25–200 µg/mL, prepared from stock solutions in 100% DMSO), and DMSO final concentration in the test solutions did not exceed 1%. Cells were exposed to samples for 24 h or 48 h, and then test solutions were removed and 100 µL of 10% (*v*/*v*) Alamar Blue^®^ solution were added to each well. The absorbance was read, at 570 nm and 620 nm, after a 5 h incubation, using a microplate reader (Multiskan EX; MTX Lab Systems, Inc., Bradenton, FL, USA). Alamar Blue^®^ reduction was calculated as described in Andreani et al. [53], and results were expressed as percentage of cell viability, in relation to untreated control. The DMSO’s final concentration in the extracts solutions was lower than 1% (*v*/*v*), at the highest concentration tested. Previous results showed that, at 1% (*v*/*v*), DMSO induced no cytotoxicity. The concentration of extracts needed to inhibit 50% of cell growth/proliferation (or the cytotoxic concentration at which 50% viability is achieved), the IC_50_, was calculated as previously describe Silva et al. [54], and is presented as mean ± S.D. (*n* = 3 independent experiments, with quadruplicates in each experiment). The extracts’ activity against the studied cell lines was categorized according to previously established criteria by the National Cancer Institute (NCI) guidelines [55]: highly active IC_50_ < 0.02 mg/mL; moderately active IC_50_ 0.02–0.20 mg/mL; weakly active IC_50_ 0.20–0.50 mg/mL and inactive IC_50_ > 0.50 mg/mL.

### 3.5. Statistical Analysis

Statistical analysis to find significant factor effects and significant differences between means of different treatments (*p* < 0.05), was performed using IBM SPSS Statistics 25 and GraphPad Prism 9 software. Shapiro–Wilk’s test was used to test normality of data and Levene’s test to test homogeneity of variance. ANOVA and post hoc Tuckey’s test were used for normal data with homogeneous variance. Welch’s ANOVA, Welch’s *t*-test, and Dunnet’s T3 post hoc test were used for normal data with equal variances not assumed. Four parameter symmetric sigmoid curves were fitted as the regression dose–response model for cell viability data (as % in relation to the control) versus extracts concentration, to extract IC_50_ values. Top and bottom plateau parameters of the sigmoid curves were fixed at 100 and 0%, because of the use of sample blanks and of a control.

## 4. Conclusions

From this work, we conclude that the Andalusian process of labdanum resin extraction rendered higher labdanum resin yields than the Zamorean process. In addition, the two resins showed different chemical profile by GC-MS analysis, which indicates the loss of relevant bioactive compounds during the Zamorean process. Labdane-type diterpenoid acids, and methylated flavonoid aglycones, already reported in the literature, were identified using the standards (mostly in the Andalusian labdanum resin), but several compounds remained unidentified for both resins. Using relevant enzymatic inhibition assays to confirm traditional medicinal uses, such as hyperglycemia control and the treatment of mental disorders, labdanum resin, mainly that extracted by Andalusian process, is worth to be further study as a medicinal product or a source of pharmaceuticals ingredients with anti-diabetic and neuroprotective activities, as the labdane-type diterpenoids revealed potential as α-amylase inhibitors, and the overall Andalusian labdanum resin revealed potential as a source of acetylcholinesterase inhibitors. Given the high cytotoxic activity against Caco-2 and HepG2 cells, the gastrointestinal and hepatic toxicity is worth being considered for labdanum resin and its products. However, this cytotoxic/anti-proliferative activity may also indicate that the labdanum diterpenoids and flavonoids are potential cytostatic compounds that are worth exploring for pharmaceutical applications as, according to NCI, these extracts are moderately active (IC_50_ 0.02–0.20 mg/mL) as anti-cancer agents.

## Figures and Tables

**Table 1:** Dry labdanum resin yield in relation to the initial plant material fresh weight and dry labdanum absolute yield in relation to the dry resin weight.

Extraction Process	Resin Yield (% dw/fw)	Absolute Yield (% dw/dw)
Adl	5.79 ± 0.63 ^a^	72.61 ± 1.52 ^a^
Zam	0.23 ± 0.08 ^b^	71.03 ± 4.18 ^a^

Values are presented as mean ± standard deviation (*n* = 3). Different coefficient letters mean statistical differences by *t*-test, with Welch’s correction to compare resin yields (α < 0.05).

**Table 2:** GC-EI-MS relevant peaks in the chromatograms of the labdanum absolutes and fractions extracted (Figures S1–S4), relative quantification in relation to the major peak area (+: 2–10%; ++: 10–50%; +++: 50–90%; M: 90–100%), tentative identification based on Kovats retention index (RI) and fragmentation *m*/*z*^-^ ions pattern compared to NIST database (Match), and identification using standards (grey).

Experimental								NIST Library/Standard Identification		
Peak	RT (min)	RI (i.u.)	Extract				TMS (73)	NIST Match	Compound	NIST RI
					Adl Fractions					
			Adl	Zam	Flv	Dit				
1	7.46 ± 0.02	1133 ± 1		+			yes	886 ± 48	3-acetoxy-3-hydroxypropionic acid, methyl ester	
2	9.64 ± 0.03	1286 ± 2	+	++			yes	921 ± 48	3-phenylpropanoic acid (hydrocinnamic acid), TMS	1279/1246
3	9.72 ± 0.02	1291 ± 1		++			yes	814 ± 78	3-methyl-hexanedioic acid, dimethyl ester	1285/1253
4	10.37 ± 0.00	1341 ± 0		+					-	
5	12.42 ± 0.10	1499 ± 8		++					-	
6	13.36 ± 0.02	1571 ± 2		++				872 ± 15	4-hydroxy-α-methyl-benzenepropanol (rhododendrol)	1585
7	14.20 ± 0.07	1630 ± 4		++					-	
8	14.98 ± 0.13	1681 ± 8		+++			yes		-	
9	16.02 ± 0.28	1742 ± 16		M			yes		-	
10	18.58 ± 0.02	1875 ± 1		+			yes		-	
11	19.80 ± 0.03	1932 ± 1		++			yes	885 ± 18	Hexadecanoic acid, methyl ester	1926/1909
12	24.58 ± 0.04	2135 ± 2		++	+	+	yes	884 ± 40	Octadecanoic acid, methyl ester	2128/2110
13	26.48 ± 0.02	2212 ± 1	++				yes		-	
14	26.59 ± 0.04	2216 ± 1	+						-	
15	26.81 ± 0.04	2225 ± 2	+						-	
16	27.00 ± 0.04	2233 ± 2	M	++	+	+	yes	862 ± 32	Labd-8(20)-en-15-oic acid, methyl ester	
17	27.21 ± 0.05	2242 ± 2	+++	++		+	yes		-	
18	27.51 ± 0.04	2254 ± 2	+					-	-	
19	27.91 ± 0.05	2269 ± 2	+++	++	+	+	yes		-	
20	29.65 ± 0.04	2339 ± 1	++	+		+	yes	837 ± 56	Eicosanoic acid, methyl ester	2329/2310
22	31.08 ± 0.01	2395 ± 0	+		+	M	yes		Labdanolic acid, TMS (standard)	
23	32.87 ± 0.04	2467 ± 1	++	++	+	++	yes	6-oxo-labd-7-en-15-oic acid/6-oxocativic acid, TMS (standard)		
24	33.46 ± 0.01	2492 ± 0	+				yes		-	
25	33.83 ± 0.01	2506 ± 0	+						-	
26	34.11 ± 0.00	2515 ± 0	+				yes		-	
27	34.49 ± 0.05	2528 ± 2	+			+	yes		-	
28	34.62 ± 0.05	2532 ± 2	+				yes	884 ± 1	Docosanoic acid, methyl ester	
29	38.39 ± 0.03	2696 ± 2	+				yes	-	-	
30	38.97 ± 0.03	2723 ± 2		++			yes	826 ± 4	Octadecanoic acid, 2,3-hydropropyl ester	
31	39.99 ± 0.00	2767 ± 0	+						-	
32	43.72 ± 0.00	2934 ± 0			++			Apigenin-4′,7-dimethyl ether		
33	44.40 ± 0.06	2963 ± 0			+++			Kaempferol-4′,3,7-trimethyl ether (methyljaranol)		
34	45.40 ± 0.01	*			+			Apigenin-4′-methyl ether (acacetin)		
35	45.66 ± 0.01	*			++			Apigenin-7-methyl ether (genkawin)		
36	46.10 ± 0.03	*	++	+	M			Kaempferol-3,7-dimethyl ether (Jaranol)		
37	47.83 ± 0.01	*			++			Apigenin		

Retention time (RT), experimental Kovats RI, and NIST Match presented as mean values ± standard deviation of all the extracts. Relative quantification based of the mean values of triplicates for each extract. * Retention time higher than for triacontane (C30).

**Table 3:** Inhibition of α-amylase activity (%) by the labdanum absolutes and fractions added at 1 and 0.5 mg/mL.

Sample	α-Amylase Inhibition (%)	
	1 mg/mL	0.5 mg/mL
Adl *	19.77 ± 0.95 ^b^	12.50 ± 1.58 ^b^
Zam	n.a.	n.a.
Dit *	39.99 ± 2.77 ^a^	29.82 ± 0.92 ^a^
Flv *	3.43 ± 0.10 ^c^	4.60 ± 0.10 ^c^

Values presented as mean ± standard deviation (*n* = 3). Dit and Flv are fractions of Adl absolute. Different coefficient letters mean statistical differences between values within each column by the post hoc Dunnett’s T3 test (*p* < 0.05). Symbol (*) means a statistical difference between the two tested concentrations for each extract by *t*-test with Welch’s correction (α = 0.05). n.a. means no activity compared to the control.

**Table 4:** Inhibition of α-glucosidase activity (%) by the labdanum absolutes and fractions added at 1 and 0.5 mg/mL.

Sample	α-Glucosidase Inhibition (%)	
	1 mg/mL	0.5 mg/mL
Adl	14.02 ± 0.87 ^a^	13.15 ± 2.17 ^abc^
Zam *	24.13 ± 4.62 ^a^	13.66 ± 0.51 ^ab^
Dit	6.43 ± 1.37 ^b^	4.34 ± 0.58 ^cd^
Flv	15.39 ± 0.94 ^a^	11.78 ± 5.28 ^abc^

Value presented as mean ± standard deviation (*n* = 3). Dit and Flv are fractions of Adl absolute. Different coefficient letters mean statistical differences between values within each column by the post hoc Dunnett’s T3 test (*p* < 0.05). Symbol (*) means a statistical difference between the two tested concentrations for each extract by *t*-test with Welch’s correction (α = 0.05).

**Table 5:** Inhibition of acetylcholinesterase activity (%) by the labdanum absolutes and fractions added at 1 and 0.5 mg/mL.

Sample	Acetylcholinesterase Inhibition (%)	
	1 mg/mL	0.5 mg/mL
Adl *	75.08 ± 0.86 ^a^	69.76 ± 0.58 ^a^
Zam	21.22 ± 6.87 ^bcd^	7.57 ± 1.29 ^cd^
Dit *	35.25 ± 0.44 ^bd^	24.08 ± 0.45 ^bc^
Flv	22.94 ± 2.64 ^cd^	22.91 ± 5.12 ^bcd^

Value presented as mean ± standard deviation (*n* = 3). Dit and Flv are fractions of Adl absolute. Different coefficient letters mean statistical differences between values within each column by the post hoc Dunnett’s T3 test (*p* < 0.05). Symbol (*) means a statistical difference between the two tested concentrations for each extract by *t*-test with Welch’s correction (α = 0.05).

**Table 6:** IC_50_ (µg/mL) of labdanum absolutes and fractions in relation to HepG2 and Caco-2 cell viability at 24 h and 48 h incubation.

Sample	HepG2			Caco-2		
	24 h		48 h	24 h		48 h
Adl	77.42 ± 3.90 ^bC^	#	48.27 ± 1.25 ^aB^	52.84 ± 4.27 ^aB^	#	36.73 ± 2.00 ^aA^
Zam	>200.00		>200.00	>200.00		>200.00
Dit	60.29 ± 2.27 ^aB^		53.77 ± 4.08 ^aB^	44.58 ± 4.51 ^aA^		38.67 ± 2.01 ^aA^
Flv	76.73 ± 2.97 ^bC^	#	52.84 ± 1.23 ^aA^	69.50 ± 2.11 ^bB^	#	48.30 ± 2.67 ^bA^

Value presented as mean ± standard deviation (*n* = 3). Dit and Flv are fractions of Adl absolute. Different coefficient small or capital letters mean statistical differences between mean values within each column or row, respectively, by the post hoc Tuckey’s test (α = 0.05). # means statistical differences between incubation times by the post hoc Tukey’s test (α = 0.05).

## Data Availability

The original contributions presented in the study are included in the article/Supplementary Material, further inquiries can be directed to the corresponding author/s.

## Supplementary Materials

The following supporting information can be downloaded at: https://www.mdpi.com/article/10.3390/molecules29102222/s1, Figure S1: GC-EI-MS chromatograms of the TMCS + BSTFA derivatized absolute of the labdanum resin obtained by the Andalusian process (peak numbers according to Table 2). Figure S2: GC-EI-MS chromatograms of the TMCS + BSTFA diterpenoid fraction of the labdanum resin obtained by the Andalusian process (peak numbers according to Table 2). Figure S3: GC-EI-MS chromatograms of the TMCS + BSTFA flavonoid fraction of the labdanum resin obtained by the Andalusian process (peak numbers according to Table 2). Figure S4: GC-EI-MS chromatograms of the TMCS + BSTFA derivatized absolute of the labdanum resin obtained by the Zamorean process (peak numbers according to Table 2). Figure S5: Four-parameter symmetric sigmoidal curves of HepG2 cell viability, as percentage in relation to the control (0.00 µg/mL), versus Adl labdanum absolute and fractions concentration at 24 h and 48 h incubation (regression parameters in Table S1). Figure S6: Four-parameter symmetric sigmoidal curves of Caco-2 cell viability, as percentage in relation to the control (0.00 µg/mL), versus Adl labdanum absolute and fractions concentration at 24 h and 48 h incubation (regression parameters in Table S1). Table S1: R-squared and Hill slope of the best fitted symmetric sigmoidal curves to the HepG2, and Caco-2 cell viability versus labdanum absolutes, and its Flv and Dit fraction, concentration data at 24 h and 48 h incubation.
