# Supplementary material for: Labdanum Resin from Cistus ladanifer L. as a Source of Compounds with Anti-Diabetic, Neuroprotective and Anti-Proliferative Activity
Source: Molecules. 2024 May 9;29(10):2222. doi: 10.3390/molecules29102222 (PMC11124373; doi:10.3390/molecules29102222)
Supplement: Supplementary file 1 [file molecules-29-02222-s001.zip › molecules-2981446-supplementary.pdf]

molecules

MDPI

Supplementary material

Labdanum Resin from *Cistus ladanifer* L. as a Source of Compounds with Anti-Diabetic, Aeuroprotective and Anti-Proliferative Activity

David F. Frazão [1,2,3], Carlos Martins-Gomes [1], Teresa Sosa Díaz [4], Fernanda Delgado [2,5], José C. Gonçalves [2,5] and Amélia M. Silva [1,6*]

1 Center for Research and Technology of Agro-Environmental and Biological Sciences (CITAB-UTAD), University of Trás-os-Montes e Alto Douro (UTAD), Quinta de Prados, 5001-801 Vila Real, Portugal; davidmfrazao@gmail.com (D.F.F.); camgomes@utad.pt (C.M.-G.)
2 Plant Biotechnology Center of Beira Interior (CBPBI), Quinta da Senhora de Mércules, Apartado 119, 6001-909 Castelo Branco, Portugal; fdelgado@ipcb.pt (F.D.); jcgoncalves@ipcb.pt (J.C.G.)
3 Mediterranean Institute for Agriculture, Environment and Development (MED) & Global Change and Sustainability Institute (CHANGE), CEBAL, 7801-908 Beja, Portugal
4 Department of Plant Biology, Ecology and Earth Sciences, Faculty of Science, University of Extremadura, 06006 Badajoz, Spain; tesosa@unex.es
5 Research Centre for Natural Resources, Environment and Society (CERNAS), Polytechnic Institute of Castelo Branco (IPCB), Quinta da Senhora de Mércules, Apartado 119, 6001-909 Castelo Branco, Portugal;
6 Department of Biology and Environment, School of Life Sciences and Environment, UTAD, Quinta de Prados; 5001-801 Vila Real, Portugal
* Correspondence: : amsilva@utad.pt (A.M.S.); Tel.: +351-259-350-921 (A.M.S.)

Contents:

Figure S1. GC-EI-MS chromatograms of the TMCS + BSTFA derivatized absolute of the labdanum resin obtained by the Andalusian process (peak numbers according to Table 2).

Figure S2. GC-EI-MS chromatograms of the TMCS + BSTFA diterpenoid fraction of the labdanum resin obtained by the Andalusian process (peak numbers according to Table 2),

Figure S3. GC-EI-MS chromatograms of the TMCS + BSTFA flavonoid fraction of the labdanum resin obtained by the Andalusian process (peak numbers according to Table 2).

Figure S4. GC-EI-MS chromatograms of the TMCS + BSTFA derivatized absolute of the labdanum resin obtained by the Zamorean process (peak numbers according to Table 2).

Figure S5. Four-parameter symmetric sigmoidal curves of HepG2 cell viability, as percentage in relation to the control (0.00 µg/mL), versus Adl labdanum absolute and fractions concentration at 24 h and 48 h incubation (regression parameters in Table S1).

Figure S6. Four-parameter symmetric sigmoidal curves of Caco-2 cell viability, as percentage in relation to the control (0.00 µg/mL), versus Adl labdanum absolute and fractions concentration at 24 h and 48 h incubation (regression parameters in Table S1).

Table S1: R-squared and Hill slope of the best fitted symmetric sigmoidal curves to the HepG2, and Caco-2 cell viability versus labdanum absolutes, and its Flv and Dit fraction, concentration data at 24 h and 48 h incubation.

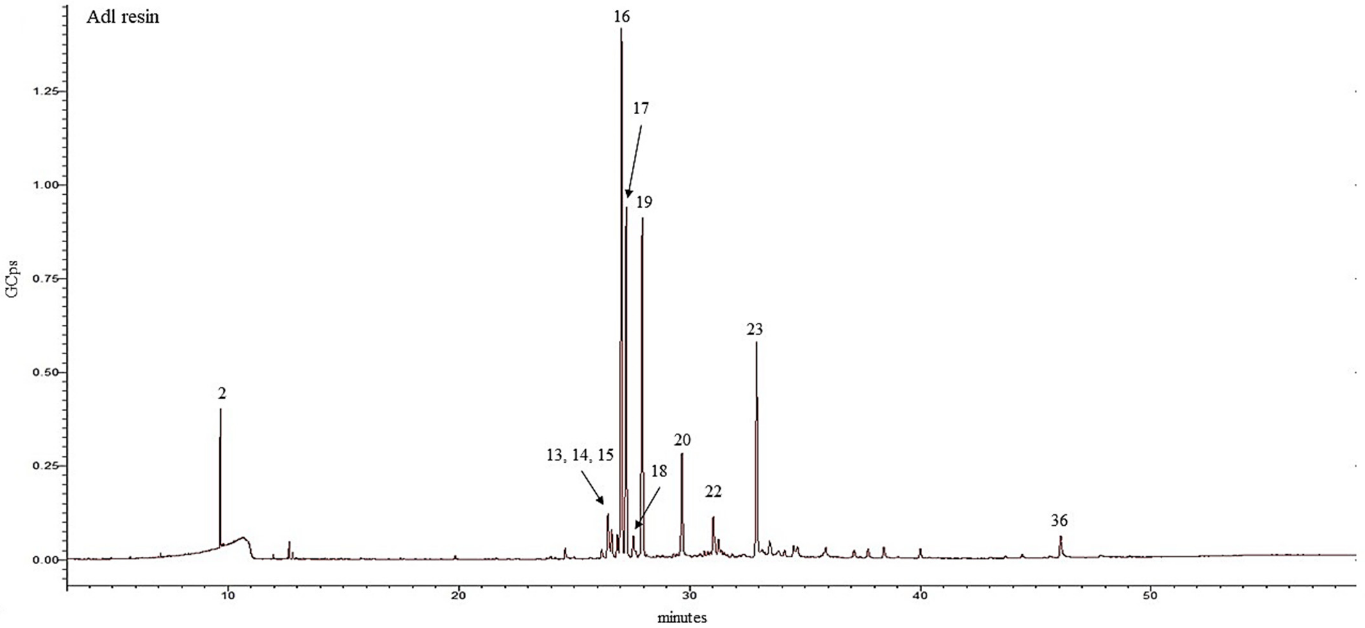

Figure S1. GC-EI-MS chromatograms of the TMCS + BSTFA derivatized absolute of the labdanum resin obtained by the Andalusian process (peak numbers according to Table 2).

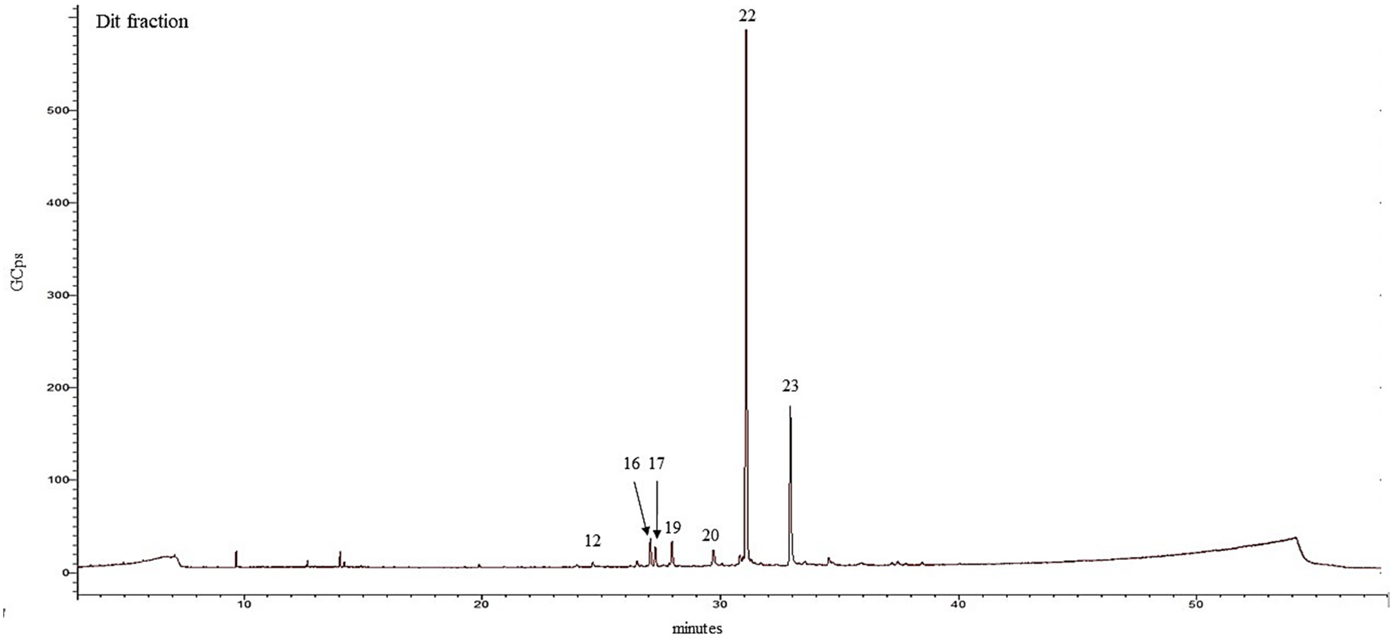

Figure S2. GC-EI-MS chromatograms of the TMCS + BSTFA diterpenoid fraction of the labdanum resin obtained by the Andalusian process (peak numbers according to Table 2).

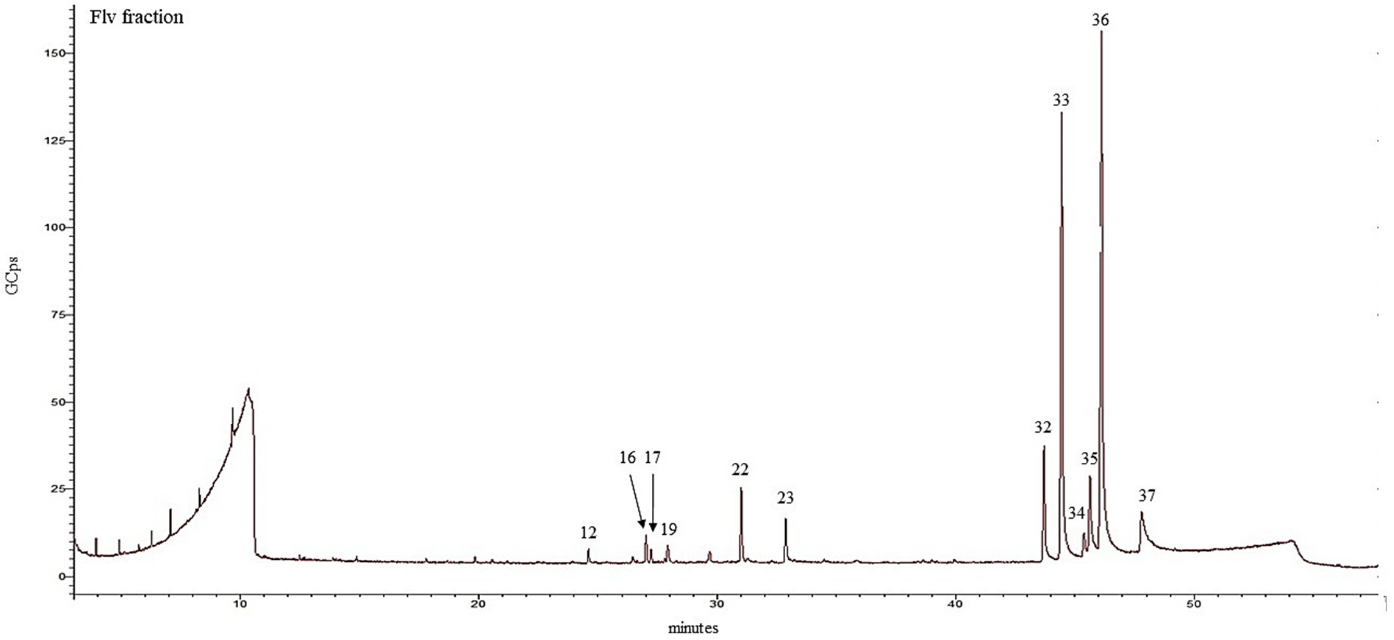

Figure S3. GC-EI-MS chromatograms of the TMCS + BSTFA flavonoid fraction of the labdanum resin obtained by the Andalusian process (peak numbers according to Table 2).

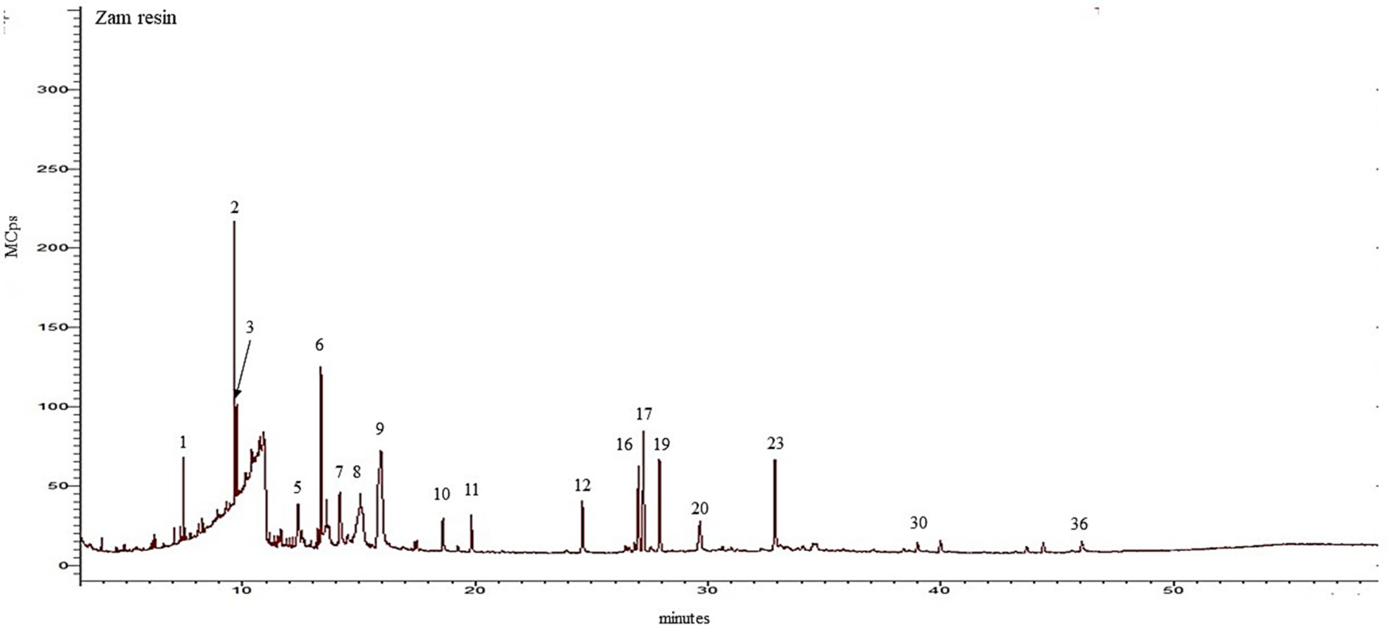

Figure S4. GC-EI-MS chromatograms of the TMCS + BSTFA derivatized absolute of the labdanum resin obtained by the Zamorean process (peak numbers according to Table 2).

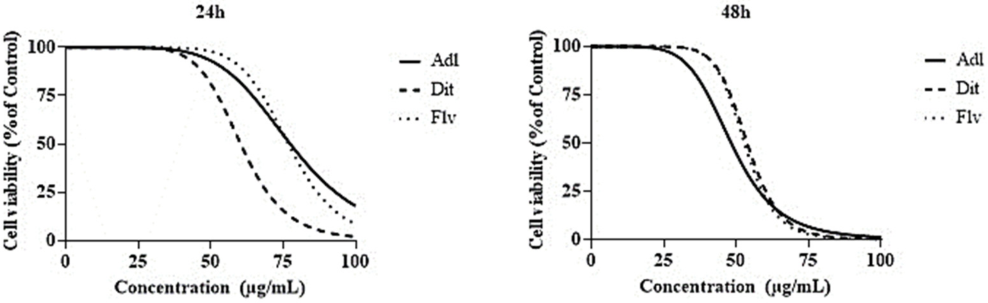

Figure S5. Four-parameter symmetric sigmoidal curves of HepG2 cell viability, as percentage in relation to the control (0.00 μg/mL), versus Adl labdanum absolute and fractions concentration at 24 h and 48 h incubation (regression parameters in Table S1).

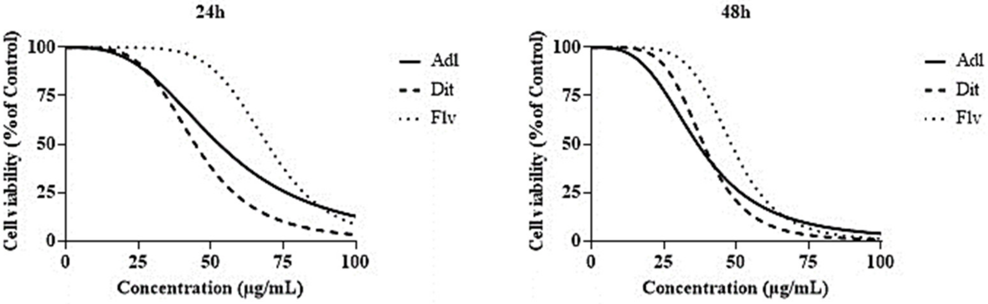

Figure S6. Four-parameter symmetric sigmoidal curves of Caco-2 cell viability, as percentage in relation to the control (0.00 μg/mL), versus Adl labdanum absolute and fractions concentration at 24 h and 48 h incubation (regression parameters in Table S1).

Table S1: R-squared and Hill slope of the best fitted symmetric sigmoidal curves to the HepG2, and Caco-2 cell viability versus labdanum absolutes, and its Flv and Dit fraction, concentration data at 24 h and 48 h incubation.

Cell	Extract	Incubation	R^2	Hill Slope
Caco-2	Adl	24 h	0.9574	-3.020 ± 0.706
		48 h	0.9883	-3.246 ± 0.419
	Dit	24 h	0.9464	-4.216 ± 1.533
		48 h	0.9904	-5.223 ± 0.849
	Flv	24 h	0.9843	-6.576 ± 1.168
		48 h	0.9755	-6.034 ± 2.667
HepG2	Adl	24 h	0.9335	-5.951 ± 1.858
		48 h	0.9942	-6.001 ± 1.221
	Dit	24 h	0.9817	-7.776 ± 1.422
		48 h	0.9178	-9.707 ± 8.163
	Flv	24 h	0.9465	-8.959 ± 3.892
		48 h	0.9934	-10.110 ± 3.440

Values presented as mean ± standard deviation (3 independent experiments of n = 4). At each data point, top and bottom plateaus were set at 100 and 0%, respectively. Zam resin did not induce cell viability reduction.
